# History, problems, and prospects of Islamic insurance (Takaful) in Bangladesh

**DOI:** 10.1186/s40064-016-2400-5

**Published:** 2016-06-18

**Authors:** Issa Khan, Noor Naemah Binti Abdul Rahman, Mohd Yakub Zulkifli Bin Mohd Yusoff, Mohd Roslan Bin Mohd Nor

**Affiliations:** Academy of Islamic Studies, University of Malaya, 50603 Kuala Lumpur, Malaysia; Department of Fiqh and Usul al-Fiqh, Academy of Islamic Studies, University of Malaya, 50603 Kuala Lumpur, Malaysia; Department of Islamic History and Civilization, Academy of Islamic Studies, University of Malaya, 50603 Kuala Lumpur, Malaysia

**Keywords:** Islamic Insurance, Problems, Regulations, Future prospects, Bangladesh

## Abstract

This study explains the history, current problems, and future possibilities of Islamic insurance (takaful) in Bangladesh. To articulate these issues, the researcher has adopted the qualitative method, and data has been collected through secondary sources i.e. articles, books, and online resources. The study reveals that Islamic insurance in Bangladesh is regulated by the Insurance Act 2010 which is contradictory with Islamic insurance causing numerous problems for Islamic insurance. This study also points out that Islamic insurance is a fast growing industry with huge prospects in Bangladesh. The government should introduce separate regulations for both Islamic and conventional insurance. The research concludes with suggestions for the further development of Islamic insurance in Bangladesh.

## Background

Islamic insurance was introduced in Bangladesh on December 12, 1999. Islami Insurance Bangladesh Ltd. is the first full fledged Islamic insurance in Bangladesh (Kalil 2011, pp. 216–217). But until now, there is no particular rule and regulation recognised by the government to regulate Islamic insurance (ARPILIL 2012, p. 2). Though several initiatives have been proposed, they have yet to be implemented for several reasons, chief among which are bureaucracy and prevailing political instability (Ali 2012). Although a series of researches have been conducted on Islamic insurance and its problems, further research is required to reach suitable solutions, particularly in the context of Bangladesh (Bekkin 2007). Few have researched insurance in Bangladesh, especially Islamic insurance (ARIIBL 2012, p.23). This article seeks to attract the attention of the Bangladesh government to introduce and implement separate regulations for Islamic insurance. It attempts to identify the problems of Islamic insurance in Bangladesh and propose solutions to further develop it in Bangladesh. A qualitative method has been adopted based on the review of different articles, books, and online resources.

### Overview of Bangladesh’s financial sector and history of insurance in Bangladesh

The financial sector plays an important role for the further development of a country (Ahmad and Malik 2009). Bangladesh’s financial sector comprises money market, credit market, capital market, Islamic and non-Islamic insurance companies, several financial institutions, and microfinance (Khan 2015, p. 106). The banking sector comprises 48 banks including 30 private commercial banks, nine foreign commercial banks, and nine nationalised commercial and specialised banks (Rahman and Ara 2009). The Central Bank of Bangladesh (Bangladesh Bank) controls and monitors all banking activities including government, non-government banks, and all other financial institutions (Masum 2012, pp. 17–18). The Securities and Exchange Commission (SEC) of Bangladesh regulates the stock market and related activities (Uddin and Suzuki 2011). The insurance sector including Islamic and non-Islamic insurance companies is regulated by the Ministry of Commerce in Bangladesh (Chowdhury 2014).

Although the genesis of insurance can be traced from ancient times, it is difficult to predict when insurance was introduced in the world civilisation (Rispler-Chaim 1991). In the Indo-Pak subcontinent, the first Insurance Act was enacted in 1938 (Samarth 2003). Insurance in Bangladesh and its organisation is based on the Insurance Act 2010 (IDAR 2013). From 1947 to 1970, several insurance companies were introduced while Bangladesh was known as East Pakistan (Rahim 2013). There were 47 insurance companies including local and foreign companies operating in East Pakistan until 1972 and the entire industry was run by the non-government companies. However, on March 26, 1972, all insurance companies, including life and general insurance, except the foreign insurance companies were nationalised by the Presidential Order titled the Bangladesh Insurance Order, 1972 (Ali 2000, pp. 22–23).

Post nationalisation of insurance companies on August 8, 1972 (Islam 1985), an ordinance promulgated by the Bangladesh government divided all insurance companies into four insurance companies and formed four corporations; two for life insurance, i.e. Rupsa Life Insurance Corporation and Surmah Life Insurance Corporation, and for general insurance business, Karnaphuli Insurance Corporation and Tista Insurance Corporation (Bachchu 2007, p. 58). Furthermore, on May 14, 1973, the provisions of the Bangladesh Insurance Corporation Ordinance were amended and the previous organisations were made into two organisations i.e. Jiban Bima Corporation and Sadharan Bima Corporation (Chowdhury and Huda 2014). These corporations dominated insurance in Bangladesh until 1984 (Samina 2012) excluding a few foreign life insurance companies. However, because of public demand and necessity for the private sector, the insurance companies were forced to authorise new insurance companies under the Insurance Ordinance 1984 (Chowdhury and Huda 2014). From 1984 until now, more than 62 private sector insurance companies have been established (Chaudhrui 2008, p. 9).

In the pre-liberation period of Bangladesh, insurance was under the authority of West Pakistan and the government did little to develop insurance (Rahim 2013) leading to limited professionally trained personnel. To develop and meet the demands of the insurance industry, the Government of the People’s Republic of Bangladesh established the Bangladesh Insurance Academy in November, 1973 (Chaudhrui 2008, p. 15). The academy offers professional certificates and diplomas, conducts research on the insurance industry, offers in-service training for officers and employees of public and private sector insurance organisations, trains insurance officers of other organisations, publishes research on insurance, establishes close contacts with local and foreign academic institutions, organises joint courses, and invites students and trainees from abroad (BIA Bangladesh Insurance Association 2016).

### Development of Islamic insurance (Takaful) in Bangladesh

The basic concept of Islamic insurance (takaful) is derived from ancient Arab tribal custom (Zainuddin and Noh 2015) whereby if anybody was murdered by a member of a different tribe the closest relative of the killer would have to compensate with blood money. This type of financial help is a protection for the heir of the deceased against the unexpected death (Billah 1980). Similarly, the practice of insurance was in existence since Islam’s earliest appearance (Swartz and Coetzer 2010). The essence of the Islamic insurance system has been reflected by the establishment of the treasury in order to help the needy Muslims and those who resided within the Islamic domain (Muhammad 2006, pp. 236–240). In the administration of the companions of Prophet Muhammad (PBUH) after his death, particularly in the time of the caliph Umar the concept of insurance clearly and obviously existed and was well organised (Hussain and Pasha 2011).

Islamic insurance during the fifth Caliph Umar ibn Abdul Aziz was also documented. He was always concerned about his subjects and used to help the poor and the needy in various ways (As-Sallabi 2011, p. 388,). In the nineteenth century, Ibn Abidin, a prominent Hanafi scholar, discussed the concept of Islamic insurance and its legality. His idea of insurance opened the mind of many Muslims who did not accept the validity of insurance practice (Sadeghi 2011). In the twentieth century, the famous Islamic jurist, Sheikh Muhammad Abduh permitted insurance practices by supporting that insurance transaction is akin to the al-Mudharabah (one kind of partnership business where one party provides the fund and other party provides labour) financing technique (Ali 2006, p. 102).

In the International Muslim Conference in Jeddah (Iqbal and Molyneux 2005, p. 190) King Faisal invoked all Muslim counties to reconstruct their banking system in the light of shariah. As a consequence of this conference, in Bangladesh, although the Islamic banking system was introduced in 1983 (Khan et al. 2008), Islamic insurance was introduced on December 12, 1999. Islami Insurance Bangladesh Ltd. is the first Islamic insurance company in Bangladesh (ARIIBL 2012, p. 5). Presently, National Life Insurance Company Ltd., Delta Life Insurance Company, Shandhani Life insurance Co. Ltd., Homeland Life insurance Co. Ltd., Magna Life Insurance Company Ltd., Rupali Life insurance Co. Ltd., Pragati Life insurance Co. Ltd., Popular Life insurance Co. and other conventional insurance companies contribute to spread and extend Islamic insurance products through opening Islamic windows (Kalil 2011, pp. 216–217). Evolvement of full pledged Islamic Insurance companies in Bangladesh is given in Table 1.

**Table 1:** Evolvement of Islamic insurance in Bangladesh Sources: ARIIBL (2012); ARTIIL (2012); ICICL (2012); FILICL (2012); ARFILICL (2012); PILIL (2012); Ali (2016)

Serial no.	Name of Islamic insurance company	Year of establishment	Branch
1	Islami Insurance Bangladesh Limited	25 October 1999	38
2	Takaful Islami Insurance Limited	21 December 1999	32
3	Islami Commercial Insurance Company Limited	01 January 2000	28
4	Padma Islami Life Insurance limited	26 April 2000	8
5	Fareast Islamic Life Insurance Company limited	29 May 2000	1012
6	Prime Islami Life Insurance limited	22 April 2002	83
7	Alpha Islami life Insurance limited	After July 2013	–
8	Trust Islami Life Insurance Company limited	After July 2013	–

### Establishment of Central *Shariah* Council for Islamic Insurance of Bangladesh

There are eight full-fledged Islamic insurance companies and 13 Islamic insurance windows operating takaful in Bangladesh (TJCSCIIB 2012, p. 5). Each takaful company and its windows have *shariah* boards to monitor takaful activities. All directors and *shariah* boards of each takaful company and its windows gathered in June 20, 2002, in order to regulate and standardise the system across all operators (ARCSCIIB 2010, p. 3). They met again in August 13, 2002 to establish the Central *Shariah* Council for Islamic insurance of Bangladesh. The Founder Chairman of this council is the Imam of the national mosque “Baitul Mukarram” Abdul Haque. The council was registered in January 19, 2008 (ARCSCIIB 2011, p. 3). The main objectives of the council are as mentioned below:

1. To regulate full *shariah* rules and regulations in each takaful company and its window.
2. To provide necessary suggestion and consultancy for takaful company and its member of *shariah* board, director-general, and board of the director.
3. To train employee and officers of each takaful company regarding Shariah compliant takaful practices and all systems related to *Shariah.*
4. Majority jurist decisions will be approved and finally this decision will be considered the decision of council.
5. The council will suggest for the each takaful company to produce each new product of takaful in the light of *Shariah.*
6. To suggest the Insurance Development & Regulatory Authority Bangladesh deal with all works related to Islamic insurance in the light of *Shariah.*
7. To collect wealth from the all takaful companies in order to spend for the needy, poor, and donate for welfare activities.
8. To arrange conferences and seminars related to takaful and suggest the government introduce separate regulations for takaful companies in Bangladesh (ARCSCIIB 2012, p. 3).

### Problems of Islamic insurance in Bangladesh

Islamic insurance throughout the world has been facing a lot of problems (Bekkin 2007). Similarly, Islamic insurance in Bangladesh has been facing numerous problems (ARTIIL 2012, p. 14). The nature of the problems is as follows:

### Lack of separate regulation, shariah-based deposit and Islamic capital market for Islamic insurance

In 2000, Bangladesh Islamic insurance companies were licensed under the Insurance Act 1938 which was not equipped to deal with Islamic insurance (Khan 2010). This is because, Islamic insurance is based on *shariah* rules and regulations (Foster 2007), while conventional insurance is based on conventional regulations (Schwarcz and Schwarcz 2014). Although a committee was formed by the government of Bangladesh to draft separate insurance laws for Islamic and conventional insurance in 2007, the outcome of the effort has yet to materialise. In 2008, the caretaker government of Bangladesh promulgated the Insurance Ordinance 2008 and the Insurance Regulatory Ordinance 2008. These were ignored by the present government (Ali 2012). Furthermore, two insurance laws were passed in the parliament of Bangladesh on March 3, 2010 which came into effect as an Insurance Act on March 18, 2010. However, it only mentioned rules for investment assets of Islamic insurance (IDRAB: Insurance Development and Regulatory Authority Bangladesh 2013). The lack of a legal and regulatory framework has stifled the Islamic insurance industry.

Islamic insurance has to deposit a large amount of money with the Central Bank of Bangladesh as security to operate whereas the Bangladesh Bank operates based on interest (*Riba*). Bangladesh Bank provides interest against this deposit, but Islamic insurance cannot receive this kind of money as a profit (Kalil 2011, pp. 218–220). Another obstacle of Islamic insurance is lack of Islamic capital market (Azad et al. 2013). Islamic insurance companies do not have any alternative for investment as without Islamic banks all bonds and certificates are based on interest. Islamic insurance companies cannot participate in this kind of investment or capital market. As a result, Islamic insurance companies lag behind conventional insurance (Kalil 2011, pp. 218–220).

### Unexpected competitions, lack of skilled people, qualified field workers and desk officers

Conventional non-government insurance companies gain illegal business through discounting or returning the premium to the policy holder. Policy holders are encouraged to dealing with conventional non-government insurance companies expecting profit or bonus at the end of the year while Islamic insurance companies do not offer any discount or return the premium to the policy holder. This is why they are less interested in an Islamic insurance policy. This kind of competition is considered a big challenge not only for Islamic insurance but also for conventional government insurance companies. Some conventional non-government insurance companies accept cheques on credit to sell policies in debt (Kalil 2011, p. 218–220). Moreover, there is a lack for skilful and experienced people in conventional insurance in Bangladesh (Reza and Iqbal 2007). In Islamic insurance, this number is even more shocking. Although 90 % of the total Bangladesh population are Muslim, there is a lack of qualified human resource in Islamic insurance (Bhuiyan et al. 2012). Furthermore, field workers and most desk officers in Islamic insurance do not enough training and Islamic education required to serve Islamic banking and insurance. This inhibits their ability to work professionally and inform the public of the benefits of their products and services (Khalid 2007, pp. 27–28).

### Lack of Islamic re-insurance and training institutions

There is no Islamic re-insurance company in Bangladesh (TJCSCIIB 2012, p. 5). Islamic insurance companies are forced to re-insure their money through conventional re-insurance. From the premium derived from these re-insurance companies at the end of the year, the conventional insurance companies distribute one part of this profit to the policy holders of the different companies as an original profit, while Islamic insurance companies cannot accept interest-based profit (Kalil 2011, pp. 218–220).

Insurance companies in Bangladesh lack training institutions for their employees. There are seven fully fledged Islamic Banks in Bangladesh. Among them only Islami Bank Bangladesh Ltd. has a training institute and opportunities to provide its employees training services. Employees are provided little training insurance (Ullah 2013). General Islamic insurance companies (general takaful companies) do not have any training institutions, although the Bangladesh Insurance Academy trains how to conduct Islamic insurance without interest but there is no opportunities for one-to-one teach training or to research the Islamic insurance system (Khalid 2007, pp. 27–28).

### Lack of public interest in Islamic insurance and consensus among muslim scholars (*Ulamah*)

Most of the people are not interested in Islamic insurance due to lack of Islamic knowledge and understanding: absence of people’s awareness, propaganda, and misinterpretation about Islamic insurance among the general people in Bangladesh (Reza and Iqbal 2007). The lack of financial solvency further reduces subscriptions to Islamic insurance. Although Bangladesh is a Muslim country, the governor is unaware of Islamic studies, especially Islamic finance (Huqe 2002, pp. 164–165). In addition, many question Islamic insurance and its operating system (Rahman 2006, p. 273) particularly in Bangladesh. Some Muslim scholars say that Islamic insurance and its operations are like conventional insurance and should be prohibited. Others argue that it is legalised by shariah through some operations remain conventional. Others encourage avoiding it completely as it is unclear whether it is truly Islamic in spirit and content (Khan 2015, p. 219). Such debates confuse the public causing them to doubt Islamic insurance.

### Proposed solutions of the problems

Steps are needed to overcome these problems. The government should introduce and implement rules and regulations specific to ensure Islamic insurance complies with the shariah. Islamic insurance can be regulated and monitored by the Bangladesh Bank, the Central Bank of Bangladesh, which can emulate Bank Negara Malaysia (Central Bank of Malaysia). Bank Negara Malaysia controls both the Islamic and conventional insurance systems through different statutory laws. Takaful is regulated by the Malaysia Takaful Act 1994 (Abu-Hussin et al. 2014). After passing this separate Act for takaful, Malaysia became a leading country for Islamic takaful (Yazid et al. 2012). To benefit from this Malaysia Takaful Act 1994, the Bangladesh government can send specialists to observe and discuss with Malaysia takaful authorities about their policy. Alternatively, it can establish a bank or institution to monitor all Islamic insurance companies in Bangladesh. This will help impose punishment by way of imprisonment, fine, or both for fraud and illegal activities.

To solve the deposit problem, the government can provide Mudarabah bonds so that Islamic insurance can buy these bonds and keep it as security (Kalil 2011, p. 218; Azad et al. 2013). Simultaneously, the government should allow establishing Islamic re-insurance companies in Bangladesh. At the same time, Islamic insurance companies should deposit money in Islamic re-insurance companies in other counties like the United Arab Emirates (Dubai) (IICUAE Islamic Insurance Companies in United Arab Emirates 2016).

Moreover, there are about 64,000 *QwmiMadrasa* (voluntary private Islamic schools not recognised by government) in Bangladesh (Ahmed 2005). They study Islamic studies based on classical Islamic books. Their syllabus includes primary level modern subjects, but no higher level reference of modern and Islamic banking, finance, and insurance. Their certificate is not also recognised by the government of Bangladesh (Ellis 2007). Madrasah students know Islamic *shariah* in depth but because of their non-recognised certificates, they cannot join any government, semi-government, and non-government sectors. If their certificates are recognised by the government and their syllabus combined with modern curriculums including modern Islamic banking and Islamic insurance, this would help meet the need for skilled employees in Islamic insurance.

Furthermore, approximately 7000 Alia Madrasa are government recognised Islamic schools in Bangladesh (Ahmad 2009, p. 32). These Madrasas can play an important role in Islamic banking, finance, and Islamic insurance by combining new syllabus with Islamic studies related to Islamic banking, finance, and insurance.

The lack of public interest can be solved using mass media. Scholars should write articles in newspapers and magazines about the negative effects of conventional insurance since it is based on interest (usury) (Hussain and Pasha 2011) and extol the benefits of Islamic insurance which is based on mutual help, brotherhood, and solidarity (Matsawali et al. 2012). Moreover, the Islami Foundation of Bangladesh has seven Imam Training Academy Centres, six divisional and 58 district offices (Islamic Foundation 2013). It can training the Imams about Islamic insurance. The trained Imams can then spread awareness of Islamic insurance and its necessity in the society. Along with this, there are 250,399 mosques in Bangladesh (Iran English Radio 2013). Imams of those mosques can play important roles advising people the benefit of Islamic Insurance. To appease pubic concerns, the government can form a National Fatwa Board comprising Muslim scholars specialised in *Fiqh al*-*Muamilat* (Islamic trade, finance, and commercial law). To this end, it can follow the policy of the Islamic Financial Service Board of Malaysia as it leads Islamic financial institutions by regulating rules and the requirements of *shariah* scholars to be a member of supervisory boards (IFSB Islamic Finance Service Bill 2012).

### Prospect of Islamic insurance (Takaful) in Bangladesh

Although the first Islamic insurance company was established in Sudan (Bekkin 2007), it has expanded globally, especially in Asia (Rahman 2013). Bangladesh is the third biggest Muslim country in the world (Rashid et al. 2008). It can be hoped that there are ample opportunities for Islamic insurance companies and grow in Bangladesh. Unfortunately, there is little published research on Islamic insurance in Bangladesh) Ali 2009, p. 3). If more articles and books would be published with media campaigns relating to Islamic insurance, these would generate greater awareness about Islamic insurance in Bangladesh.

Bangladeshis are generally religious minded and tend to avoid things that contravene with Islam including in trade and commerce (Miah 1992). In the absence of an Islamic banking system, they used to consider the conventional banking system (Sarker 1999). Later on, revolutionary changes were observed in the public mindset regarding the banking sector in Bangladesh with the establishment of Islamic banking in 1983 (Ahmed et al. 2006). If Islamic insurance is fair and transparent in their transactions, people might avoid conventional insurance (Sarwar 2016). The Fareast Life Insurance Company is a good example of the revolution of the Islamic insurance sector in Bangladesh and is a fast growing insurance company (Kalil 2011, pp. 217–218).

There are eight full-fledged Islamic insurance companies in Bangladesh. Among them, five are life Islamic insurance and three are general Islamic insurance (Ali 2016). Thirteen conventional insurance companies have opened Islamic insurance windows (Sarwar 2016). Islamic insurance has emerged as a fast growing industry in Bangladesh. Since 2004–2008, the total premium increased from Tk. 1.4 million (USD 0.02 million) to Tk. 5.7 million (USD 0.07 million) forming 12 % of total premiums in the insurance sector. Meanwhile, more than 60 Islamic insurance companies have applied for approval from the Bangladesh Insurance Academy (Ali 2006). It indicates that the demand and prospect of Islamic insurance is positively attracting interest across the country. Size and growth of insurance industry in Bangladesh is mentioned in Tables 2 and 3.

**Table 2:** Size and growth of life insurance Source: Ali (2016)

Category	Gross premium		Market share		Growth rate (%)
	2009–2010	2011–2012	2009–2010 (%)	2011–2012 (%)	
BDT million					
Islamic Insurance	8637.59	5669.30	33.02	25.95	−34.36
Insurance	17,520.09	16,178.85	66.98	74.05	−7.66
Total	26,157.68	21,848.14

**Table 3:** Size and growth of non- life insurance Source: Ali (2016)

Category	Gross premium		Market share		Growth rate (%)
	2009–2010	2011–2012	2009–2010 (%)	2011–2012 (%)	
BDT million					
Islamic Insurance	1185.18	2326.40	5.19	4.55	96.29
Insurance	21,659.95	48,769.80	94.81	95.45	125.16
Total	22,845.13	51,096.21			

## Conclusion

Islamic insurance was first introduced to Bangladesh in 1999 in Bangladesh. As yet, Islamic insurance is not properly regulated due to lack of co-operation by the government and Islamic insurance companies. To overcome this problem, the government should enact separate regulations for Islamic insurance. Nevertheless, Islamic insurance is a fast growing industry in Bangladesh with positive prospects. Finally, the proposed suggestions of the study, which are not included in the existing literatures, may contribute to solve problems of Islamic Insurance and form Islamic economy in Bangladesh.

## References

[CR1] Abu-Hussin MF, Muhamad NHN, Hussin MYM (2014). Takaful (Islamic Insurance) Industry in Malaysia and the Arab Gulf States: challenges and future direction. Asian Soc Sci.

[CR2] Ahmad M (2009) Views from the Madrasa: Islamic education in Bangladesh. The National Bureau of Asian Research NBR project

[CR3] Ahmad E, Malik A (2009). Financial Sector Development and Economic Growth: an Empirical Analysis of Developing Counties. J Eco Co-operat Develop.

[CR4] Ahmed S (2005) Testimony of Samina Ahmed to US Senate Foreign Relations Committee. http://www.crisisgroup.org/en/publication-type/speeches/2005/testimony-of-samina-ahmed-to-us-senate-foreign-relations-committee.aspx. Accessed 29 May 2013

[CR5] Ahmed E, Rahman Z, Ahmed RI (2006). Comparative analysis of loan recovery among nationalized, private and islamic commercial banks of Bangladesh. BRAC Univ J.

[CR6] Ali MAS (2000). Jiban Bima Bangladesh Preckhapot.

[CR7] Ali KMM (2006). Introduction to Islamic Insurance.

[CR8] Ali KMM (2009). Islami Jiban Bima Bartaman Pakkitha. Institute of Islamic Thought, Dhaka

[CR9] Ali KMM (2012) Bangladesh: The need for Takaful regulations. http://www.primeislamilifebd.com/Article/TheNeedTakReg. Accessed 20 Apr 2012

[CR10] Ali KMM (2016) Takaful in Bangladesh Seeking a framework for growth. http://www.primeislamilifebd.com/aritcle/takaful/Takafulinbangladesh. Accessed 5 May 2016

[CR11] ARCSCIIB (2010) Annual Report Central Shariah Council for Islamic Insurance of Bangladesh

[CR12] ARCSCIIB (2011) Annual Report Central Shariah Council for Islamic Insurance of Bangladesh

[CR13] ARCSCIIB (2012) Annual Report Central Shariah Council for Islamic Insurance of Bangladesh

[CR14] ARFILICL (2012) Annual Report Fareast Islami Life Insurance Company Ltd

[CR15] ARIIBL (2012) Annual Report Islamic Insurance Bangladesh Ltd

[CR16] ARPILIL (2012) Annual Report Prime Islamic Life Insurance Ltd

[CR17] ARTIIL (2012) Annual Report Takaful Islami Insurance Ltd

[CR18] Azad MAK, Kabir MR, Bhuiyan F, Masum AKM (2013). Prospects Analysis of an Islamic Capital Market in Bangladesh. Global J Manag Business Res Finan.

[CR19] As-Sallabi AM (2011) The Rightly-Guided Caliph and Great Reviver ‘Umar bin Abd al-Aziz. Darussalam, Riyadh

[CR20] Bachchu H (2007). Report on Performance Evaluation of ALiCO-Bangladesh.

[CR21] Bekkin RI (2007). Islamic Insurance: National Features and Legal Regulation. Arab Law Quart.

[CR22] Bhuiyan MAH, Hossain MB, Rahman MK (2012). Islamic Management Practices in Islamic Life Insurance Companies of Bangladesh. J Novel Appl Sci.

[CR23] BIA: Bangladesh Insurance Association (2016). http://www.bia.gov.bd/about-bia/objective.html. Accessed 4 May 2016

[CR24] Billah MM (1980). Islamic Insurance: its origins and development. Arab Law Quart.

[CR25] Chaudhrui AH (2008). Risk and Insurance.

[CR26] Chowdhury SE (2014) Financial System in Bangladesh. http://www.scribd.com/doc/29065718/Financial-System-in-Bangladesh. Accessed 4 May 2014

[CR27] Chowdhury TA, Huda F (2014). Performance evaluation of selected private life insurance companies in Bangladesh. Int J Business Manag.

[CR28] Ellis T (2007). Madrasas in Bangladesh. Inst Peace Conflict Studies.

[CR29] FILICL: Fareast Islami Life Insurance Company Ltd (2012). http://www.fareastislamilife.com. Accessed 20 Sep 2012

[CR30] Foster NGD (2007). Islamic Finance Law as an Emergent Legal System. Arab Law Quart.

[CR31] Huqe ABMN (2002). Ismamee Bima.

[CR32] Hussain MM, Pasha AT (2011). Conceptual and Operational Differences between General Takaful and Conventional Insurance. Aust J Business Manag Res.

[CR33] ICICL: Islami Commercial Insurance Co.Ltd (2012) http://www.islamicommercialinsurance.org/index.php.option=com_content&view=article&id=77&Itemid=677). Accessed 20 Dec 2012

[CR34] IDRAB: Insurance Development & Regulatory Authority Bangladesh (2013). http://www.idra.org.bd/idra-org/index.htm. Accessed 17 June 2013

[CR35] IFSB: Islamic Finance Service Bill (2012). http://malaysianlaw.my/attachments/Malaysian-law-DR412012E−71,326. Accessed 18 June 2013

[CR36] IICUAE: Islamic Insurance Companies in United Arab Emirates (2016). http://yp.theemiratesnetwork.com/browse/insurance_companies-islamic/united_arab_emirates/. Accessed 16 Feb 2016

[CR37] Iqbal M, Molyneux P (2005) Thirty Years of Islamic Banking: History, Performance and Prospects Palgrave Macmillan, London. Reviewed by: Abdelkader C (2006) Islamic Economics Research Centre King Abdulaziz University. J KAU: Islamic Econ. 19(1): 37–39

[CR38] Iran English Radio (2013) Bangladesh has 250,399 mosques: Minister. http://english.irib.ir/radioislam/news/islam-in-asia/item/77497-bangladesh-has-250399-mosques-minister. Accessed 10 June 2013

[CR39] Islam SS (1985). The Role of the State in the Economic Development of Bangladesh during the Mujib Regime (1972–1975). J Develop Areas.

[CR40] Islamic Foundation (2013) http://www.mora.gov.bd/?id=7. Accessed 17 Apr 2013

[CR41] Kalil ME (2011). Islamee Banking and Insurance.

[CR42] Khalid MSA (2007) Islami Economy and Insurance some Issues. Shiddikiyah Publication, Dhaka

[CR43] Khan MHUZ (2010). The Effect of Corporate Governance Elements on Corporate Social Responsibility (CSR) Reporting Empirical Evidence from Private Commercial Banks of Bangladesh. Int J Law Manag.

[CR44] Khan I (2015) Juristical Study of Takaful and it’s application in Bangladesh. Dissertation, University of Malaya

[CR45] Khan MSN, Hassan MK, Shahid AI (2008) Banking Behavior of Islamic Bank Customers in Bangladesh. J Islam Econ Bank Finance 159–194

[CR47] Masum IA (2012) An Anatomy of Central Bank’s Supervisory Functions with Special Reference To Bangladesh Bank. Dissertation, AIT (Asian Institute of Technology) School of Management

[CR48] Matsawali MS, Abdullah MF, Ping YC, Abidin SY, Zaini MM, Ali HM (2012). A Study on Takaful and Conventional Insurance Preferences: The Case of Brunei. Int J Business Social Sci.

[CR49] Miah MMR (1992). The Cultural-Structural Contexts of High Fertility in Bangladesh: a Sociological Analysis. Int Rev Modern Sociol.

[CR50] Muhammad AR (2006) Theory and Application of Islamic Insurance in Malaysia and Sudan: a Comparative Study. Dissertation, University of Malaya

[CR51] PILIL: Prime Islami Life Insurance Limited (2012). http://www.primeislamilifebd.com/service.php. Accessed 1 Sep 2012

[CR52] Rahim MO (2013). Insurance Business and Khuda Buksh’s Contributions. J Asiat Soc Bangladesh.

[CR53] Rahman FAA (2006). Insurance in Islam.

[CR54] Rahman MA (2013). Comparative Study on the Efficiency of Bangladeshi Conventional and Islamic Life Insurance Industry: a non parametric approach. Asian Business Rev.

[CR55] Rahman MM, Ara LA (2009). Trade in Financial Service in Developing Countries: a Case of the Bangladesh Financial Sector. J Int Trade Law Policy.

[CR56] Rashid M, Hassan MK, Ahmad AUF (2008). Quality Perception of the Customers towards Domestic Islamic Banks in Bangladesh. J Islamic Eco Bank Finan.

[CR57] Reza SM, Iqbal MM (2007). Life insurance marketing in Bangladesh. Daffodil Int Univ J Business Eco.

[CR58] Rispler- Chaim V (1991). Insurance and Semi-Insurance Transactions in Islamic History until the 19th Century. J Eco Social History Orient.

[CR59] Sadeghi M (2011). The Evolution of Islamic Insurance—Takaful: a literature Survey. Insurance Markets Companies Anal Actuar Comput.

[CR60] Samarth RD (2003) Transformation of Indian Insurance Industry in the 21st Century. Key Note Address at the Forty-Eighth Annual Conference, Kolkata, 7 September

[CR61] Samina QS (2012). Investment Portfolio of Insurance Companies in Bangladesh: a Study on Selected Insurance Companies of Bangladesh. World J Social Sci.

[CR62] Sarker MAA (1999). Islamic Banking in Bangladesh: performance, Problems & Prospects. Int J Islamic Finan Serv.

[CR63] Sarwar MJ (2016). Future Challenge in Islamic Insurance (Takaful) in Bangladesh. Aust J Sustainable Business Society.

[CR64] Schwarcz D, Schwarcz SL (2014). Regulating Systemic risk in insurance. Univ Chicago Law Rev.

[CR65] Swartz NP, Coetzer P (2010). Takaful: an Islamic Insurance Instrument. J Develop Agri Eco.

[CR66] TJCSCIIB (2012) Takaful Journal of Central Shariah Council for Islamic Insurance of Bangladesh

[CR67] Uddin SMS, Suzuki Y (2011). Financial Reform, Ownership and Performance in Banking Industry: the Case of Bangladesh. Int J Business Manag.

[CR68] Ullah MW (2013) Role of the Shariyah Officer (Muraqib) as Practiced by Prime Islami Life Insurance Ltd., Bangladesh

[CR69] Yazid AS, Arifin J, Hussin MR, Daud WNW (2012). Determinants of Family Takaful (Islamic Life Insurance) Demand: a Conceptual Framework for a Malaysian Study. Int J Business Manag.

[CR70] Zainuddin S, Noh INM (2015). An Overview of the Emergence of Takaful: an Islamic Type of Insurance Policy. Int J Business Eco Res.

